# Supplementary material for: Ultra-wideband optical coherence elastography from acoustic to ultrasonic frequencies
Source: Nat Commun. 2023 Aug 16;14:4949. doi: 10.1038/s41467-023-40625-y (PMC10432526; doi:10.1038/s41467-023-40625-y)
Supplement: Supplementary file 1 — Supplementary Information [file 41467_2023_40625_MOESM1_ESM.pdf]

Supplementary Materials for

Ultra-wideband optical coherence elastography from acoustic to ultrasonic frequencies

Xu Feng,[a,1] Guo-Yang Li,[a,1] and Seok-Hyun Yun[a,b,*]

[a] Harvard Medical School and Wellman Center for Photomedicine, Massachusetts General Hospital, 50 Blossom St. BAR-8, Boston, Massachusetts 02114, USA

[b] Harvard-MIT Health Sciences and Technology, Cambridge, Massachusetts 02139, USA

[1] These authors contributed equally

* Correspondence to syun@hms.harvard.edu

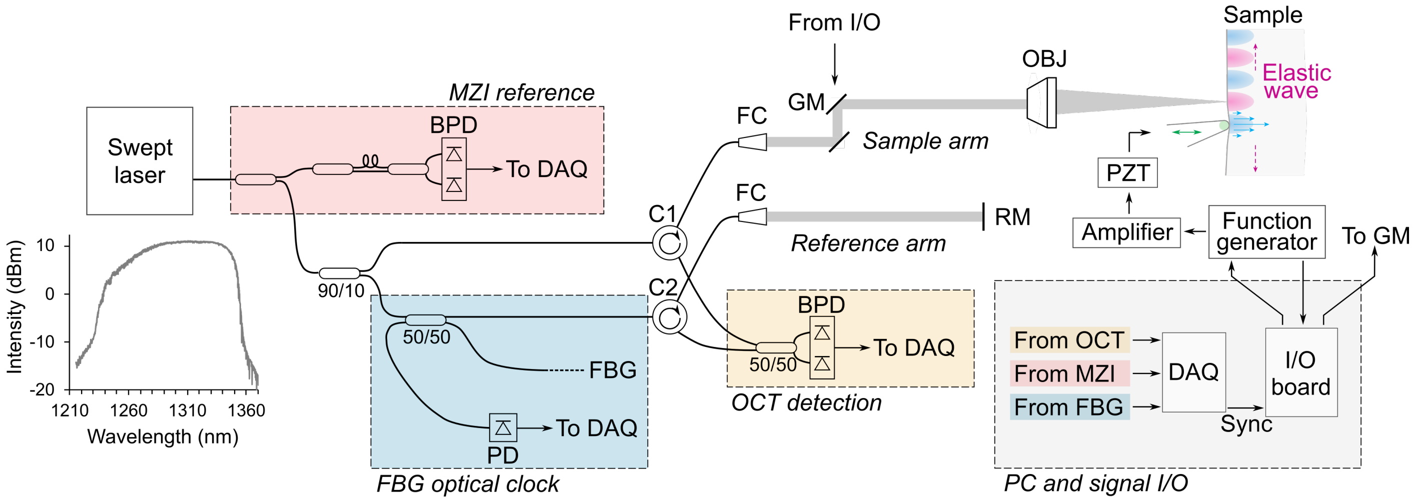

Fig. S1 Schematic of the optical imaging system and signal generation and acquisition. The output spectrum of the laser is measured by an optical spectral analyzer (AQ6370C, Yokogawa Electric). Abbreviations used: Mach-Zehnder interferometer (MZI), photodiode (PD), balanced photodiode (BPD), data acquisition (DAQ) system, Input/output (I/O) board, fiber Bragg grating (FBG), circulators (C1, C2), galvanometer mirrors (GM), fiber collimator (FC), objective lens (OBJ), reference mirror (RM), dichroic mirror (DM), piezoelectric transducer (PZT).

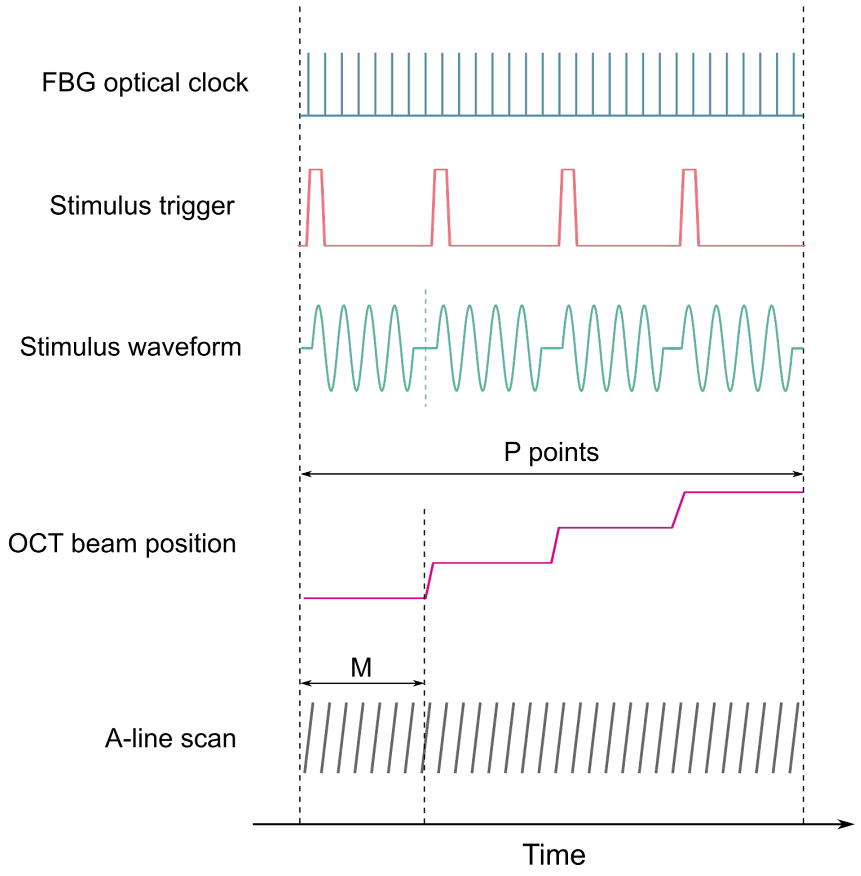

Fig. S2 Timing diagrams of the OCE system. The fiber Bragg grating (FBG) optical clock provides the sync signal for the I/O board that controls the stimulus signal, the OCT beam scan, and data acquisition. For stimulus frequencies below 21.6 kHz, the I/O board generates the harmonic stimulus directly. For stimulus frequencies above 21.6 kHz, the I/O board generates a stimulus waveform to externally trigger a function generator to send the harmonic stimulus.

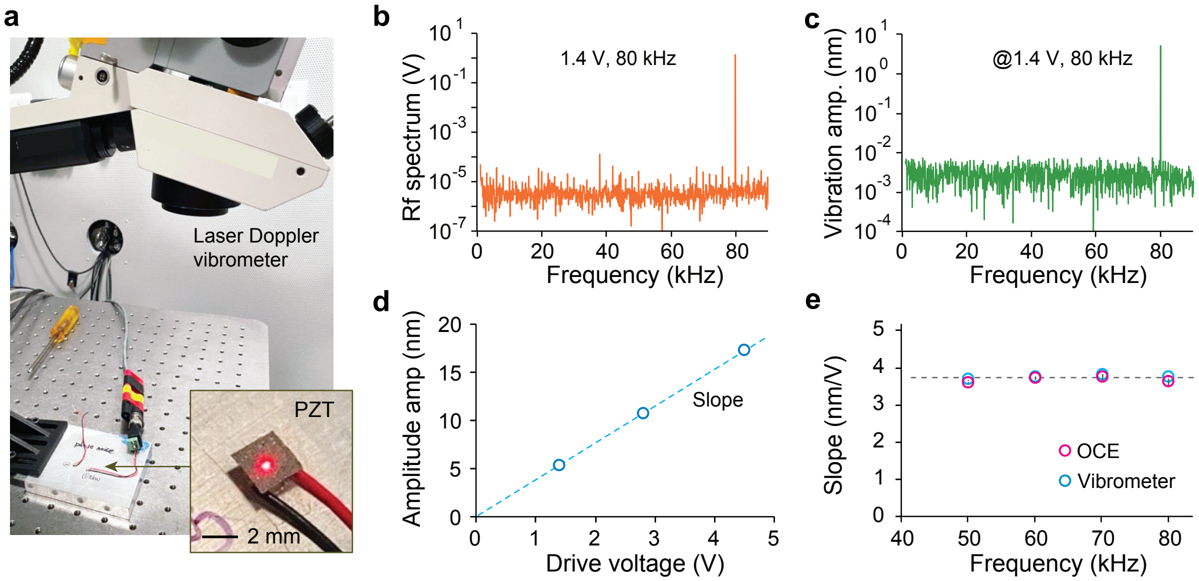

Fig. S3 Vibration amplitude measurement using a laser Doppler vibrometer. a, Experimental setup. Inset: zoom-in picture of the PZT. An adhesive retro-reflective tape was attached on the surface to enhance the vibrometer signal. **b,** The rf spectrum of a drive signal with an amplitude of 1.4 V and a frequency of 80 kHz. **c,** Measured vibrometer scan output for the given drive voltage in b. **d,** Linear slope efficiency of the PZT at 80 kHz. **e,** Comparison of the slope efficiencies measured by the laser vibrometer (red) and the OCE instrument (blue). The two datasets coincide within measurement accuracy.

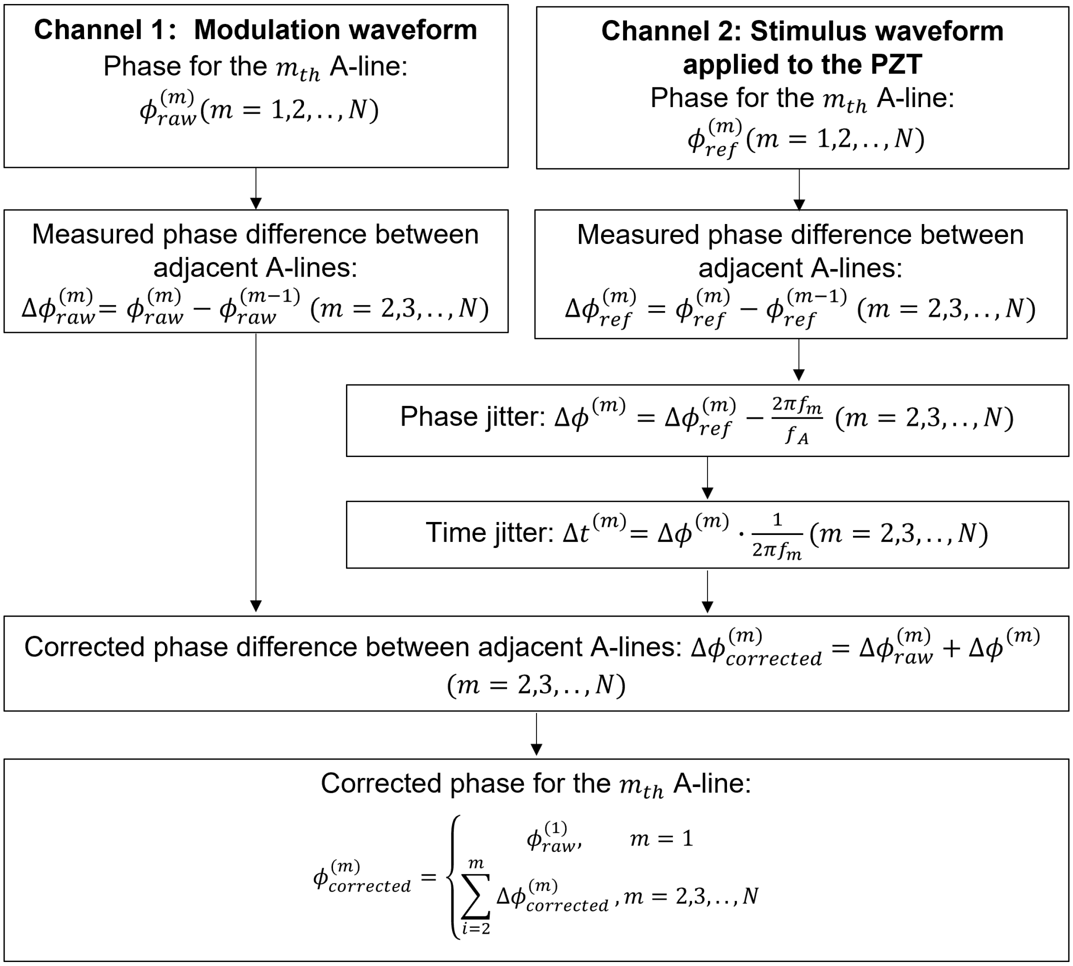

Fig. S4 Flow chart of the algorithm to correct the phase noise induced by laser time jitter. Channel 1 represents the vibration modulation waveform measured on the sample. Channel 2 represents the stimulus waveform generated by the function generator and applied to the piezoelectric transducer (PZT). The stimulus waveform applied to the PZT is recorded using a separate analog input channel while the modulation waveform is measured. Channel 1 and Channel 2 are synchronized to the same trigger related to a particular laser wavelength. Therefore, they contain the same time jitters. The phase jitter over a sweep period (i.e., a M-scan containing m A-lines) is obtained by comparing the phase of the reference waveform with the theoretical phase. f_A is the A-line rate, f_m is the vibration frequency.

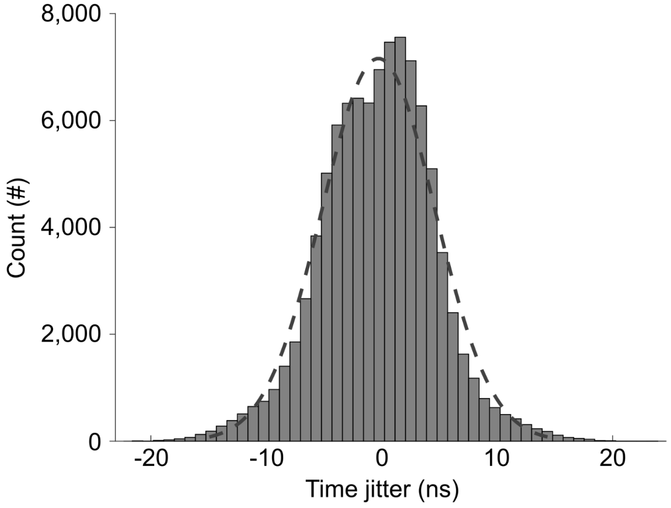

Fig. S5 Gaussian distribution of the laser time jitter. The dataset was obtained from 900 M-scans, each consisting of 108 A-lines. We have used the algorithm depicted in Fig. S4 to calculate the time jitter of each A-line. The Gaussian curve fit (dashed line) shows a standard deviation of 4.9 ns.

Supplementary Note 1. Vibration noise introduced by laser time jitter

According to Eq. (17) in the main text, the noise for the vibration phase primarily depends on the optical phase noise ($e^{-i2k_0z_0}$) and the laser time jitter ($e^{-im\omega_m T}$). The latter one comes from the mechanical instability of the swept-source laser, which results in a time jitter in the FBG clock T. For our system, we measured the standard deviation of T, denoted by $\sigma_T \approx 4.9$ ns.

Let's consider a measurement to a vibrating scatter using N continuous A scan (M-scan). The standard deviation of the vibration phase noise, denoted by σ_φ, is

$$\sigma_\varphi = \sqrt{(\sigma_{X'})^2 + (\sigma_L)^2}, \tag{S-1}$$

where σ_L denotes the phase noise introduced by laser time jitter and $\sigma_{X'}$ is the optical phase noise. $\sigma_{X'}$ is determined by the SNR of the sidelobe (denoted by X'),

$$\sigma_{X'} = \frac{1}{\sqrt{N}}\frac{1}{\sqrt{X'}}. \tag{S-2}$$

According to Eq. (16) in the main text,

$$10\log_{10}(X') = 10\log_{10}(X) + 20\log_{10}(k_0\delta), \tag{S-3}$$

where X is the SNR of the static OCT signal (main lobe).

To derive the expression for σ_L, we denote the time interval of m-th A scan with $(T + t_m)$ $(m = 1,2,..,N)$. Therefore, the mean and standard deviation of t_m are 0 and σ_T, respectively. Then the time of the m-th A scan, denoted by T_m, is $T_m = \sum_{i=1}^{m}(T + t_i) = mT + \sum_{i=1}^{m} t_i$. The vibration signal, according to Eq. (17) in the main text, is $F_m \sim e^{i\omega_m T_m}$. We perform Fourier transform to this signal to get

$$\mathcal{F}(\omega) \sim \sum_{m=1}^{N} e^{i\omega_m T_m} e^{-\omega m T} = \sum_{m=1}^{N} e^{im(\omega_m - \omega)T} e^{i\omega_m(\sum_{i=1}^{m} t_i)}. \tag{S-4}$$

At $\omega = \omega_m$, we have

$$\mathcal{F}(\omega_m) \sim \sum_{m=1}^{N} e^{i\omega_m(\sum_{i=1}^{m} t_i)} \approx \sum_{m=1}^{N}[1 + i\omega_m(\sum_{i=1}^{m} t_i)]. \tag{S-5}$$

The phase of $\mathcal{F}(\omega_m)$, denoted by $\arg\{\mathcal{F}(\omega_m)\}$, is

$$\arg\{\mathcal{F}(\omega_m)\} \approx \frac{\omega_m \sum_{m=1}^{N}(\sum_{i=1}^{m} t_i)}{N} = \frac{\omega_m}{N}[Nt_1 + (N-1)t_2 + \cdots + t_N]. \tag{S-6}$$

Then σ_L is the standard deviation of $\arg\{\mathcal{F}(\omega_m)\}$,

$$\sigma_L \approx \omega_m \sqrt{\left(\frac{N}{N}\right)^2 \left(\sigma_{t_1}\right)^2 + \left(\frac{N-1}{N}\right)^2 \left(\sigma_{t_2}\right)^2 + \cdots + \left(\frac{1}{N}\right)^2 \left(\sigma_{t_N}\right)^2}. \tag{S-7}$$

where $\sigma_{t_m} = \sigma_T$ is the standard deviation of t_m $(m = 1,2,..,N)$. Therefore, we get

$$\sigma_L \approx \frac{\omega_m \sigma_T}{N}\sqrt{\frac{N(N+1)(2N+1)}{6}} \approx \sqrt{\frac{N}{3}}(\omega_m \sigma_T). \tag{S-8}$$

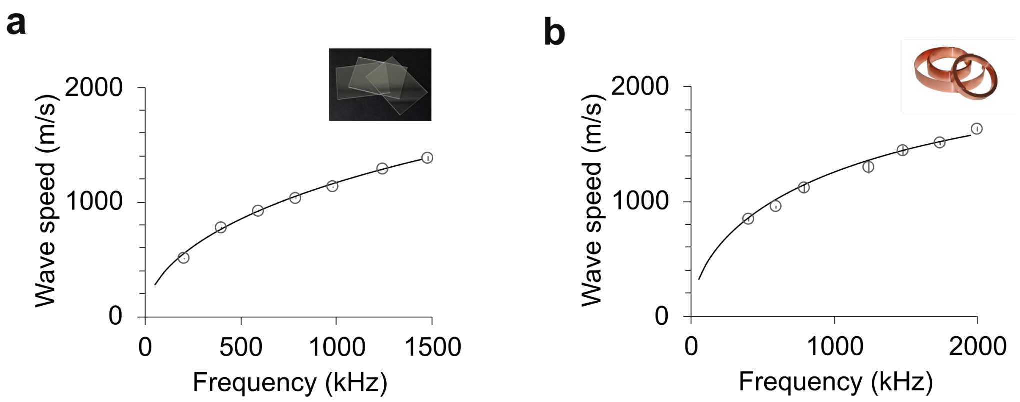

Fig. S6 Ultrasonic OCE of the glass and metal. a, Dispersion relation of the coverslip glass (dot) and theoretical fitting with a Lamb wave model (line). **b**, Dispersion relation of the copper sheet (dot) and the theoretical fitting with a Lamb wave model (line).

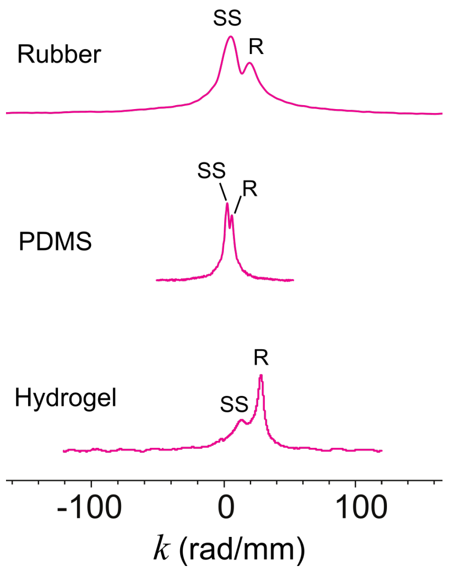

Fig. S7 Wavenumber k domain plots obtained by the Fourier Transform. Comparison is made at 40 kHz. The Rayleigh surface wave mode (R) and the supershear surface wave (SS) mode are labeled on the plots.

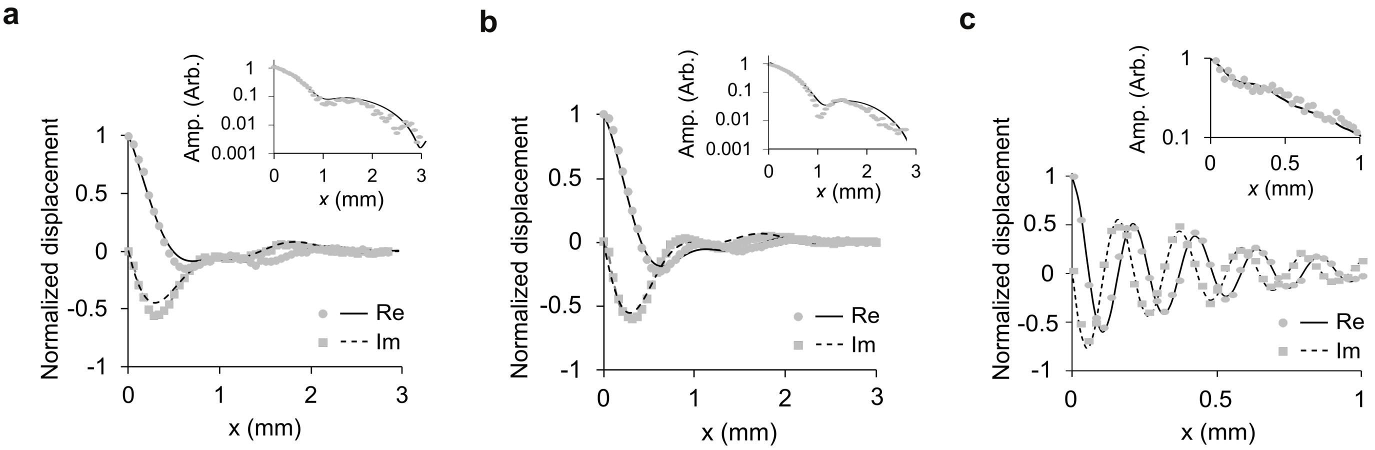

Fig. S8. Rheological fitting of the soft materials. a, Rubber (20 kHz). **b**, PDMS (40 kHz). **c**, Hydrogel (40 kHz). A comparison is maded between the experimental data and the theoretical model for the surface displacement. Markers, experiment; lines, theory. Inset: the total wave amplitude.

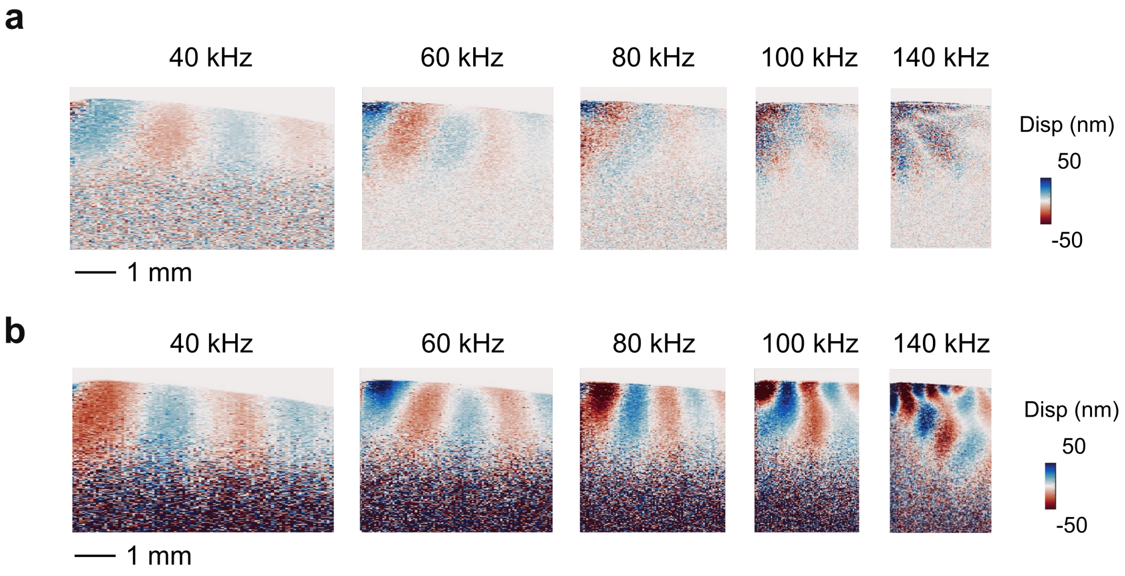

Fig. S9. Cartilage OCE. Cross-sectional displacement images at 40 – 140 kHz for **a**, Without applying the demodulation algorithm for inner scatters, and **b**, After applying the demodulation algorithm for inner scatterers.

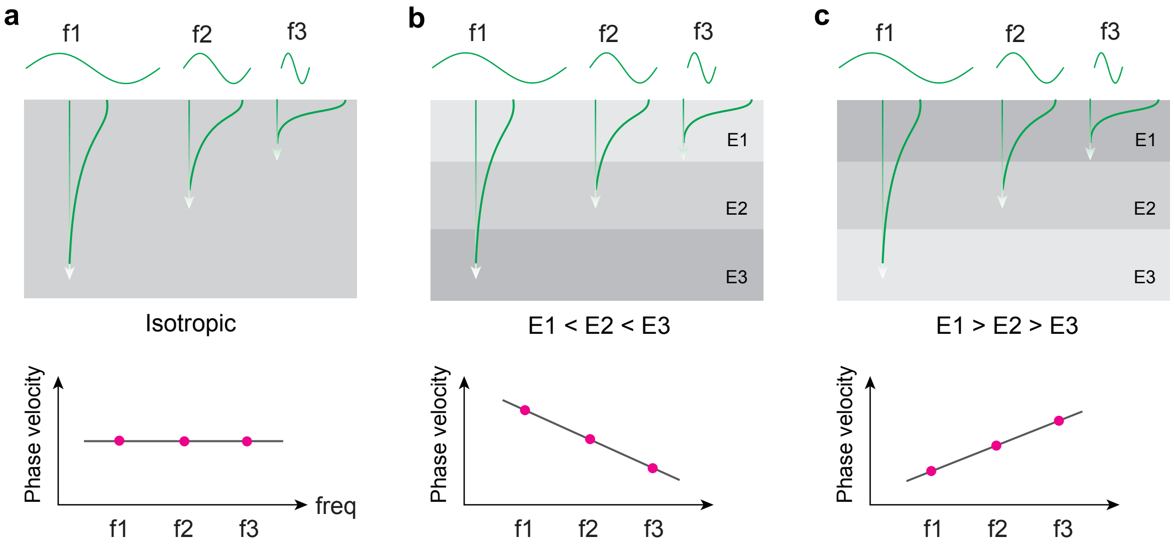

Fig. S10 Penetration of Rayleigh surface waves. a, Isotropic material. **b**, Multilayer material with softer top and stiffer bottom (e.g., the cartilage). **c,** Multilayer material with stiffer top and softer bottom (e.g., the skin). E1, E2, and E3 represent the elastic moduli of the three layers. Lower graphs represent dispersion curves. In isotropic materials, the phase velocity is independent of the stimulus frequencies (f1, f2, and f3). For multilayer materials, the phase velocity varies with frequency as the penetration depth of the Rayleigh wave varies with frequency. The amplitude of Rayleigh wave is reduced to 1/e is at a depth of about a half wavelength.

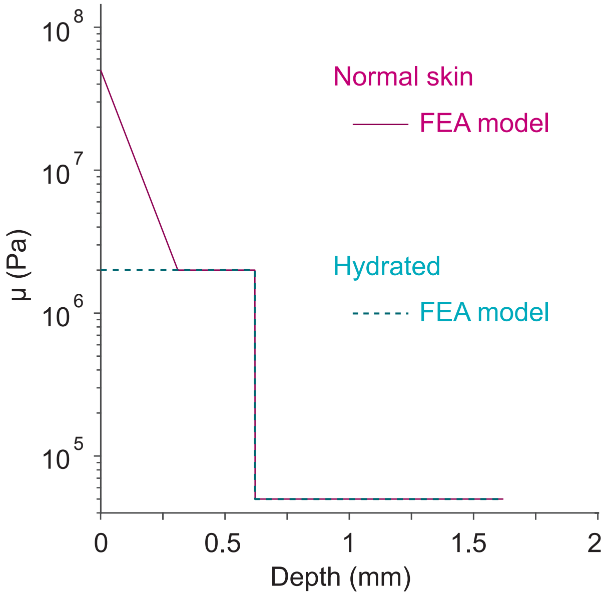

Fig. S11 FEA model for the skin. The depth dependent profile of shear modulus in the human skin for the normal, pre-hydrated state (margenta) and hydrated state (turquoise).
