# Supplementary material for: Ultra-wideband optical coherence elastography from acoustic to ultrasonic frequencies
Source: Nat Commun. 2023 Aug 16;14:4949. doi: 10.1038/s41467-023-40625-y (PMC10432526; doi:10.1038/s41467-023-40625-y)
Supplement: Supplementary file 3 — Reporting Summary [file 41467_2023_40625_MOESM3_ESM.pdf]

nature portfolio

Corresponding author(s): Seok-Hyun Yun

Last updated by author(s): Jul 17, 2023

Reporting Summary

Nature Portfolio wishes to improve the reproducibility of the work that we publish. This form provides structure for consistency and transparency in reporting. For further information on Nature Portfolio policies, see our Editorial Policies and the Editorial Policy Checklist.

Statistics

For all statistical analyses, confirm that the following items are present in the figure legend, table legend, main text, or Methods section.

n/a	Confirmed	
☐	☒	The exact sample size (*n*) for each experimental group/condition, given as a discrete number and unit of measurement
☐	☒	A statement on whether measurements were taken from distinct samples or whether the same sample was measured repeatedly
☒	☐	The statistical test(s) used AND whether they are one- or two-sided<br>*Only common tests should be described solely by name; describe more complex techniques in the Methods section.*
☒	☐	A description of all covariates tested
☒	☐	A description of any assumptions or corrections, such as tests of normality and adjustment for multiple comparisons
☐	☒	A full description of the statistical parameters including central tendency (e.g. means) or other basic estimates (e.g. regression coefficient) AND variation (e.g. standard deviation) or associated estimates of uncertainty (e.g. confidence intervals)
☒	☐	For null hypothesis testing, the test statistic (e.g. F, t, r) with confidence intervals, effect sizes, degrees of freedom and P value noted<br>*Give P values as exact values whenever suitable.*
☒	☐	For Bayesian analysis, information on the choice of priors and Markov chain Monte Carlo settings
☒	☐	For hierarchical and complex designs, identification of the appropriate level for tests and full reporting of outcomes
☒	☐	Estimates of effect sizes (e.g. Cohen's *d*, Pearson's *r*), indicating how they were calculated

Our web collection on statistics for biologists contains articles on many of the points above.

Software and code

Policy information about availability of computer code

Data collection	Data were collected from a dual-balanced photodetector (Thorlabs, PDB110C, 100 MHz) and digitized by data acquisition board (Signatec, PX14400, 14 bit) integrated in the custom swept-source OCT imaging system. Custom code was scripted in MATLAB R2019a to coordinate data acquisition. Custom codes generated in this study are deposited under DOI link: https://zenodo.org/record/8148674.
Data analysis	Data were analyzed by Microsoft Excel (Version 2306 Build 16.0.16529.20100, Microsoft Inc.), and Matlab R2019a (MathWorks, Inc.) software. Finite element analysis was performed with Abaqus 6.12 (Dassault Systèmes).

For manuscripts utilizing custom algorithms or software that are central to the research but not yet described in published literature, software must be made available to editors and reviewers. We strongly encourage code deposition in a community repository (e.g. GitHub). See the Nature Portfolio guidelines for submitting code & software for further information.

Data

Policy information about availability of data

All manuscripts must include a data availability statement. This statement should provide the following information, where applicable:

- Accession codes, unique identifiers, or web links for publicly available datasets
- A description of any restrictions on data availability
- For clinical datasets or third party data, please ensure that the statement adheres to our policy

The authors declare that all data supporting the findings of this study are available within the article and the supplementary information. The source data are deposited under DOI link: https://doi.org/10.6084/m9.figshare.23162636. Any additional data are available from the corresponding author.

Research involving human participants, their data, or biological material

Policy information about studies with human participants or human data. See also policy information about sex, gender (identity/presentation), and sexual orientation and race, ethnicity and racism.

Reporting on sex and gender	One 31-year-old male subject was recruited.
Reporting on race, ethnicity, or other socially relevant groupings	One Asian subject was recruited.
Population characteristics	We recruited one subject in total. Due to the small sample size, there are potential bias in the measured mechanical properties which vary from person to person. However, as our major purpose is to demonstrate the feasibility of the proposed technique in measuring broadband data, having just one subject does not affect our results.
Recruitment	The study was conducted at the Massachusetts General Hospital (MGH) following approval from the Institutional Review Board (IRB) of Massachusetts General Hospital and the Mass General Brigham Human Research Office. The subject was recruited in response to the recruitment email sent to the Wellman employees. Written informed consent was obtained from both subjects prior to the measurement. All methods were performed in accordance with the relevant guidelines and regulations. The clinical trial is registered at www.clinicaltrials.gov (National Clinical Trial Identifier: NCT03230981).
Ethics oversight	This study was approved by the Instituional Review board of Massachusetts General Hospital and Mass General Brigham Human Research Office.

Note that full information on the approval of the study protocol must also be provided in the manuscript.

Field-specific reporting

Please select the one below that is the best fit for your research. If you are not sure, read the appropriate sections before making your selection.

[x] Life sciences [ ] Behavioural & social sciences [ ] Ecological, evolutionary & environmental sciences

For a reference copy of the document with all sections, see nature.com/documents/nr-reporting-summary-flat.pdf

Life sciences study design

All studies must disclose on these points even when the disclosure is negative.

Sample size	The sample size for our study is N = 1. Given our specific objective of showcasing the capability of our technique to measure broadband data, we did not conduct a formal sample size calculation. Consequently, having just one subject is deemed sufficient for our purposes.
Data exclusions	No data were excluded.
Replication	All the wave speed measurements in this study were repeated three times or more independently with similar results.
Randomization	The allocation of the sample was not randomized in this study. However, randomization is not deemed relevant for the purpose of demonstration for the capability of our technique in measuring broadband data.
Blinding	The group allocation was not blinded in this study. However, blinding is not deemed relevant to our study because we only recruited one subject for demonstrating the capability of our technique in measuring broadband data.

Reporting for specific materials, systems and methods

We require information from authors about some types of materials, experimental systems and methods used in many studies. Here, indicate whether each material, system or method listed is relevant to your study. If you are not sure if a list item applies to your research, read the appropriate section before selecting a response.

Materials & experimental systems

n/a	Involved in the study
☒	☐ Antibodies
☒	☐ Eukaryotic cell lines
☒	☐ Palaeontology and archaeology
☒	☐ Animals and other organisms
☐	☒ Clinical data
☒	☐ Dual use research of concern
☒	☐ Plants

Methods

n/a	Involved in the study
☒	☐ ChIP-seq
☒	☐ Flow cytometry
☒	☐ MRI-based neuroimaging

Clinical data

Policy information about clinical studies

All manuscripts should comply with the ICMJEguidelines for publication of clinical research and a completedCONSORT checklist must be included with all submissions.

Clinical trial registration	NCT03230981
Study protocol	2017P000867
Data collection	Data were collected on 6/16/2022 in MGH BHX 612 lab. Total recruitment time was 1 hour.
Outcomes	The primary outcome was assessed by non-invasive optical imaging using our lab's custom-built instrument. Wave speeds before and after the hydration test were compared. We did not use secondary outcome measures.
